# HAS (Hajibandeh Index, American Society of Anesthesiologists Status, and Sarcopenia) Model Versus NELA (National Emergency Laparotomy Audit) Score in Predicting the Risk of Mortality After Emergency Laparotomy: A Retrospective Cohort Study

**DOI:** 10.7759/cureus.50180

**Published:** 2023-12-08

**Authors:** Saranya Linganathan, Ioan Hughes, Alwin Puthiyakunnel Saji, Kalyan Mitra, Shahin Hajibandeh, Shahab Hajibandeh

**Affiliations:** 1 Department of General Surgery, University Hospital of Wales, Cardiff, GBR; 2 School of Medicine, Cardiff University, Cardiff, GBR; 3 General Surgery, Royal Stoke University Hospital, Stoke-on-Trent, GBR

**Keywords:** nela, has, model, mortality, laparotomy

## Abstract

Background

The National Emergency Laparotomy Audit (NELA) mortality risk score is currently used in the UK to estimate mortality risk after emergency laparotomy. The HAS (Hajibandeh Index, American Society of Anesthesiologists status, and sarcopenia) is a novel model with excellent accuracy in predicting the risk of mortality after emergency laparotomy. This study aimed to compare the predictive performance of the HAS model and NELA score in estimating mortality risk following emergency laparotomy.

Methodology

A retrospective cohort study was conducted including consecutive adult patients who underwent emergency laparotomy between January 2019 and January 2022. Thirty-day mortality was the primary outcome. In-hospital mortality and 90-day mortality were the secondary outcomes. The predictive tools were compared in terms of discrimination via receiver operating characteristic curve analysis, calibration via the Hosmer-Lemeshow test, and classification via classification table.

Results

Analysis of 818 patients showed that the area under the curve of HAS was superior to NELA for 30-day mortality (0.97 vs. 0.86, p < 0.0001), in-hospital mortality (0.90 vs. 0.83, p = 0.0004), and 90-day mortality (0.90 vs. 0.83, p = 0.0004). HAS demonstrated good calibration for 30-day mortality (p = 0.286), in-hospital mortality (p = 0.48), and 90-day mortality (p = 0.48) while NELA score showed poor calibration for 30-day mortality (p = 0.001), in-hospital mortality (p = 0.001), and 90-day mortality (p = 0.001).

Conclusions

The HAS model was superior to the NELA score in predicting mortality after emergency laparotomy. The HAS model may be worth paying attention to for external validation.

## Introduction

The National Emergency Laparotomy Audit (NELA) mortality risk score is currently used in the UK to estimate mortality risk after emergency laparotomy [1]. Although the discriminative power of the NELA score has been good with the area under the curve (AUC) ranging between 0.80 and 0.89, it has never demonstrated excellent discrimination [2-6]. On the other hand, the NELA score has been criticized for being less accurate for older and frail patients, for underestimation or overestimation of mortality risk, and for not taking into account the modern predictors of mortality [2-6].

In our recent study, we developed and validated an emergency laparotomy mortality risk predictive model called HAS, which included the following three components: Hajibandeh Index (HI), American Society of Anesthesiologists (ASA) status, and sarcopenia [7]. The model was developed after a very strict multivariable analysis which included the following variables: HI, ASA status, sarcopenia, age ≥80 years, clinical frailty scale (CFS), presence of intraperitoneal contamination, and need for bowel resection [7]. The predictive performance of the HAS model in estimating the risk of 30-day mortality after emergency laparotomy was excellent in terms of discrimination (AUC = 0.96), classification, and calibration [7].

This study aimed to compare the predictive performance of the HAS model and NELA score in estimating mortality risk following emergency laparotomy.

## Materials and methods

Reporting and ethical standards

The Health Research Authority and Health and Care Research Wales approved the protocol of this study via the Integrated Research Application System (IRAS ID: 320962). The design and conduct of this study were compliant with the Strengthening the Reporting of Cohort Studies in Surgery guideline for observational studies [8].

Study design and patient selection

The study was conducted in a tertiary general surgery center at a teaching hospital in South Wales. The design was a retrospective cohort with a prospective data collection approach. Consecutive adult patients with non-traumatic abdominal pathology undergoing emergency laparotomy between January 2019 and January 2022 were included. The prospectively maintained hospital electronic medical record system was used to identify the eligible patients and for data collection.

Primary and secondary outcomes

Thirty-day postoperative mortality was the primary outcome. It was defined as mortality due to any cause within 30 days after emergency laparotomy. The secondary outcomes were in-hospital mortality and 90-day postoperative mortality. In-hospital mortality was defined as mortality due to any cause during hospital stay, and 90-day postoperative mortality was defined as mortality due to any cause within 90 days after emergency laparotomy.

The risk prediction tools

HAS Model

The HAS model included the following three components: HI, ASA status, and sarcopenia [7]. The HI was calculated using the formula described in our previous articles including C-reactive protein (CRP), neutrophils, and lactate as nominators (their levels increase in abdominal sepsis) and albumin and lymphocytes as denominators (their levels decrease in abdominal sepsis) [9]. The ASA status was defined and classed as per the ASA Physical Status classification system [10]. Psoas muscle index (PMI) adjusted based on each patient’s height (mm^2^/m^2^) was used to measure sarcopenia based on the age and sex-specific cut-off values reported by Kim et al. [11]. The cross-sectional area of both right and left psoas muscles at the level of the bottom of the L3 vertebral body on the 0.625 mm thick axial abdominal CT scan was calculated using the picture archiving and communication system in our center (FUJIFILM Medical Corp. Ltd., Tokyo, Japan. Software: Synapse V5.7.240.16413) [7].

NELA Risk Score

The NELA model includes age, gender, ASA status, preoperative laboratory tests, the Glasgow coma scale score, systolic blood pressure, heart rate, cardiac and respiratory signs, operative severity, intraoperative blood loss, peritoneal soiling, severity of malignancy, and urgency of surgery [12].

Data collection

The prospectively maintained electronic hospital records were used as the source for data collection. An electronic data collection sheet was created for data collection. The following data items for each patient were collected: age, gender, ASA status, indication for laparotomy, the procedure performed, need for bowel resection, presence of peritoneal contamination, CFS, sarcopenia, mortality outcomes, NELA mortality risk, and HAS mortality risk.

Statistical analyses

The MedCalc 13.0 software was used for statistical analyses. The demographics, clinical characteristics, and outcome data were summarized with mean ± standard deviation (SD) or median and interquartile range (IQR) for continuous variables, and percentages for categorical variables. The discrimination of the models was compared using the receiver operating characteristic (ROC) curve analysis by calculating the AUC of each model. The calibration of the models was evaluated using the Hosmer-Lemeshow test (goodness of fit test), and the classification of the models was evaluated using a classification table with a cut-off value of 0.5. Two-tailed statistical tests with a 95% confidence level were applied.

## Results

Baseline characteristics of the included population

**Figure 1:**
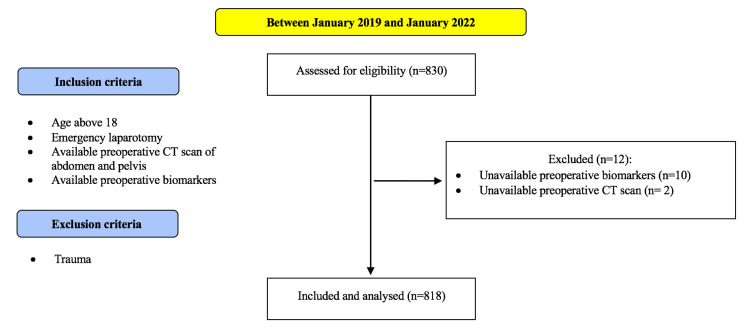
The study flow diagram.

A total of 830 patients underwent emergency laparotomy due to non-traumatic abdominal pathology between January 2019 and January 2022; 12 patients were excluded due to unavailable preoperative biomarkers (10 patients) or preoperative CT scans (two patients). Consequently, 818 patients were included for analysis. The study flowchart is shown in Figure 1. Ninety-day follow-up data were available for all patients. The mean age of the included patients was 61 years (95% confidence interval (CI) = 60-62) and 15% were ≥80 years old. In terms of sex, 50% were male and 50% were female. Overall, 7% of patients were classed as ASA I, 37% as ASA II, 41% as ASA III, 14% as ASA IV, and 1% as ASA V. Bowel resection was required in 53% of patients, and 26% had peritoneal contamination. The median CFS was 2 (IQR = 1-3), and sarcopenia was present in 10% of patients. The baseline characteristics of the included patients are summarized in Table 1.

**Table 1:** Baseline characteristics of the included patients. CI = confidence interval; ASA = American Society of Anesthesiologists; IQR = interquartile range

Number of patients	818
Age, mean (95% CI)	61 (60–62)
Age ≥80	125 (15%)
Male, n (%)	409 (50%)
Female, n (%)	409 (50%)
ASA, n (%)	
I	61 (7%)
II	300 (37%)
III	337 (41%)
IV	114 (14%)
V	6 (1%)
Indication for laparotomy, n (%)	
Small bowel obstruction	323 (40%)
Large bowel obstruction	119 (15%)
Perforated peptic ulcer	41 (5%)
Small bowel perforation	30 (4%)
Colonic perforation	131 (16%)
Intestinal ischemia	41 (5%)
Intra-abdominal collection	25 (3%)
Colitis	48 (6%)
Anastomotic leak	26 (3%)
Other	34 (4%)
Need for bowel resection, n (%)	431 (53%)
Intraperitoneal contamination, n (%)	213 (26%)
Clinical Frailty Scale, median (IQR)	2 (1–3)
Hajibandeh Index, median (IQR)	12.5 (1.7–71.6)
Sarcopenia, n (%)	83 (10%)
30-day mortality, n (%)	57 (7%)
In-hospital mortality, n (%)	77 (9%)
90-day mortality, n (%)	77 (9%)

Postoperative mortality

The risks of 30-day mortality, in-hospital mortality, and 90-day mortality were 7%, 9%, and 9%, respectively.

Performance of the HAS model versus the NELA score for 30-day mortality

Discrimination

**Figure 2:**
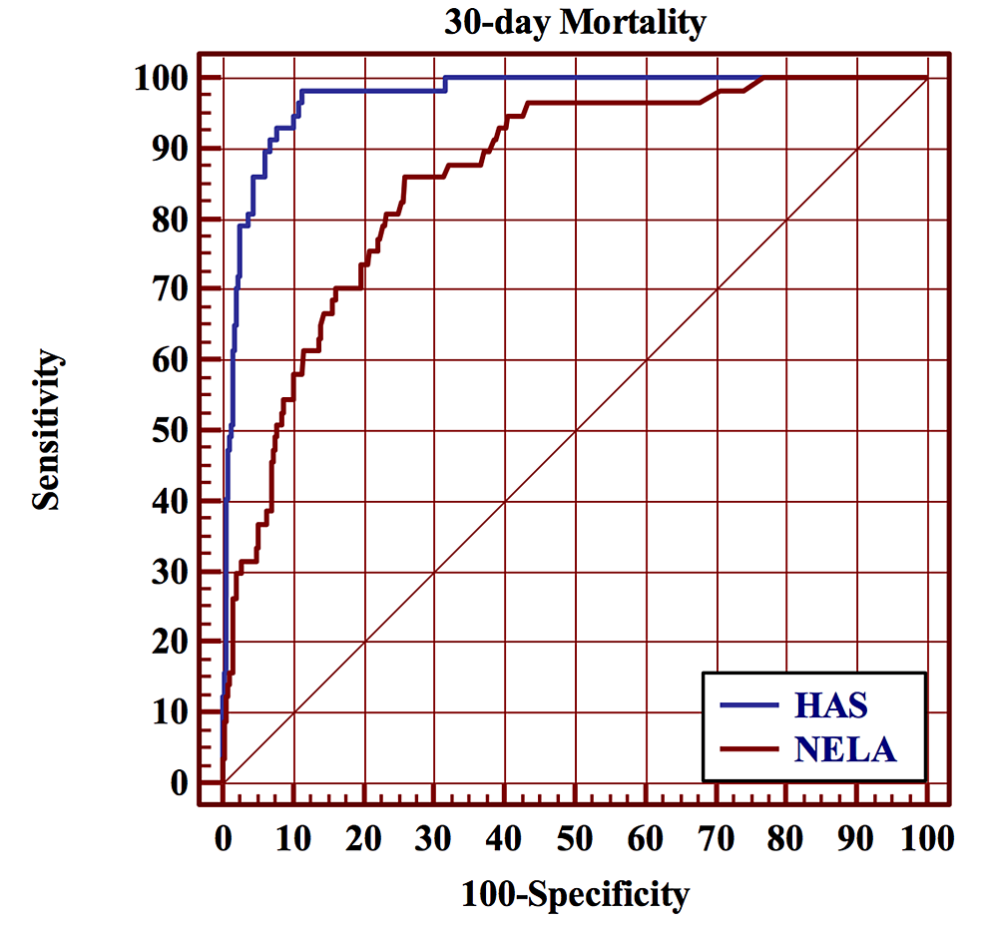
Results of the ROC curve analysis for the comparison of the HAS model (AUC = 0.97 (95% CI 0.96-0.98)) and NELA score (AUC = 0.86 (95% 0.83-0.87)) in predicting 30-day postoperative mortality. ROC = receiver operating characteristic; HAS = Hajibandeh Index, American Society of Anesthesiologists status, and sarcopenia; NELA = National Emergency Laparotomy Audit; AUC = area under the curve; CI = confidence interval

The discriminative power of the HAS model (AUC = 0.97 (95% CI = 0.96-0.98)) was significantly better (p < 0.0001) than the NELA score (AUC: 0.86 (95% 0.83-0.87)) (Figure 2).

Calibration

The Hosmer-Lemeshow test confirmed good calibration of the HAS model (p = 0.286) but poor calibration of the NELA score (p = 0.001).

Classification

The HAS model and NELA score correctly classified 95% and 92% of cases, respectively.

Secondary Outcomes

**Figure 3:**
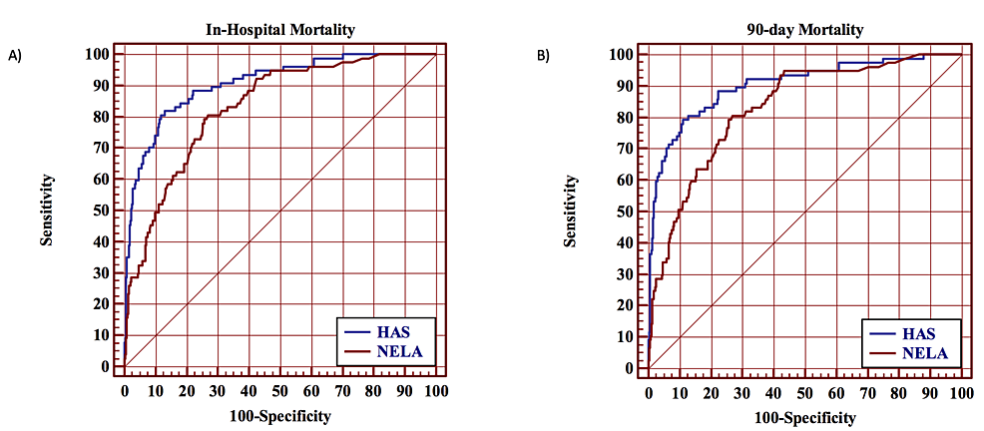
(A) ROC curve analysis for the comparison of the HAS model and NELA score in predicting in-hospital and (B) 90-day postoperative mortality. ROC = receiver operating characteristic; HAS = Hajibandeh Index, American Society of Anesthesiologists status, and sarcopenia; NELA = National Emergency Laparotomy Audit

The discriminative power of the HAS model was significantly better than the NELA score in predicting in-hospital mortality (AUC = 0.90 vs. 0.83, p = 0.0004) and 90-day mortality (AUC = 0.90 vs. 0.83, p = 0.0004) (Figure 3). HAS demonstrated good calibration for in-hospital mortality (p = 0.48) and 90-day mortality (p = 0.48) while NELA score showed poor calibration for in-hospital mortality (p = 0.001) and 90-day mortality (p = 0.001). The HAS model and NELA score correctly classified 93% and 91% of cases, respectively.

## Discussion

In our previous study, we developed and validated an emergency laparotomy mortality risk predictive model called HAS with promising results [7]. In this study, we compared the performance of the HAS model with the NELA score which is currently used in the UK. The analysis of 818 patients showed that the HAS model was superior to the NELA score in predicting postoperative mortality after emergency laparotomy in terms of discrimination and calibration.

The better performance of the HAS model can be explained by several factors. Each component of the HAS model is a strong predictor of postoperative mortality on its own [7,9,13-16]. It uses HI to take into account the severity of abdominal pathology [9]. Peritoneal contamination, tissue necrosis, or intestinal ischemia are associated with elevated levels of lactate, neutrophil, and CRP (nominator of HI) and decreased levels of lymphocyte and albumin (denominator of HI); hence, the more severe the underlying sepsis due to abdominal pathology, the higher the HI. On the other hand, the HAS model uses ASA status and sarcopenia to take into account the physical status of the patient in terms of comorbidities, frailty, and physiological reserve [13-16]. Based on the available evidence, there is no doubt that sarcopenia is a predictor of mortality in patients undergoing emergency laparotomy and many authors recommended that they should be included in preoperative risk assessment tools. Ming et al. [17] conducted a retrospective analysis of 500 patients and supported combining ASA classification with sarcopenia in risk assessment scores.

The HAS is the first preoperative predictive model that demonstrated excellent performance in predicting the risk of 30-day mortality after emergency laparotomy [7]. The performance of the HAS model is promising; however, it needs to be externally validated by other researchers. The HAS mortality risk calculator can be used to externally validate the performance of the HAS model [18]. The results of external validation by other researchers would help establish whether or not the HAS model can be incorporated into routine practice.

This study has a few limitations. The retrospective nature of the study would subject the results to the inevitable risk of selection bias. As the study was a single-center study, the generalizability of findings should be done with caution. We excluded 12 patients due to unavailable perioperative data; however, considering that the sample size of the study was relatively large, we do not believe the aforementioned exclusion affected our findings.

## Conclusions

The HAS model was superior to the NELA score in predicting mortality after emergency laparotomy. The HAS model may be worth paying attention to for external validation. The HAS mortality risk calculator is available for external validation.
